# Carbon Nanotube- and Carbon Fiber-Reinforcement of Ethylene-Octene Copolymer Membranes for Gas and Vapor Separation

**DOI:** 10.3390/membranes4010020

**Published:** 2014-01-03

**Authors:** Zuzana Sedláková, Gabriele Clarizia, Paola Bernardo, Johannes Carolus Jansen, Petr Slobodian, Petr Svoboda, Magda Kárászová, Karel Friess, Pavel Izak

**Affiliations:** 1 Institute of Chemical Process Fundamentals of the AS CR, Rozvojová 135, 165 02 Prague 6, Czech Republic; E-Mails: sedlakova@icpf.cas.cz (Z.S.); karaszova@icpf.cas.cz (M.K.); izak@icpf.cas.cz (P.I.); 2 Institute on Membrane Technology, ITM-CNR, Via P. Bucci 17/C, 87036 Rende (CS), Italy; E-Mails: g.clarizia@itm.cnr.it (G.C.); p.bernardo@itm.cnr.it (P.B.); 3 Department of Polymer Engineering, Faculty of Technology, Tomas Bata University in Zlin, Nam, TGM 275, 762 72 Zlin, Czech Republic; E-Mails: slobodian@ft.utb.cz (P.Sl.); svoboda@ft.utb.cz (P.Sv.); 4 Centre of Polymer Systems, University Institute, Tomas Bata University in Zlin, Nad Ovcirnou 3685, 760 01 Zlin, Czech Republic; 5 Department of Physical Chemistry, Institute of Chemical Technology, Technická 5, 160 00 Prague 6, Czech Republic; E-Mail: karel.friess@vscht.cz

**Keywords:** poly(ethylene-*co*-octene), carbon fibers, carbon nanotubes, mixed matrix membrane, membrane separation, transport properties, mechanical properties

## Abstract

Gas and vapor transport properties were studied in mixed matrix membranes containing elastomeric ethylene-octene copolymer (EOC or poly(ethylene-*co*-octene)) with three types of carbon fillers: virgin or oxidized multi-walled carbon nanotubes (CNTs) and carbon fibers (CFs). Helium, hydrogen, nitrogen, oxygen, methane, and carbon dioxide were used for gas permeation rate measurements. Vapor transport properties were studied for the aliphatic hydrocarbon (hexane), aromatic compound (toluene), alcohol (ethanol), as well as water for the representative samples. The mechanical properties and homogeneity of samples was checked by stress-strain tests. The addition of virgin CNTs and CFs improve mechanical properties. Gas permeability of EOC lies between that of the more permeable PDMS and the less permeable semi-crystalline polyethylene and polypropylene. Organic vapors are more permeable than permanent gases in the composite membranes, with toluene and hexane permeabilities being about two orders of magnitude higher than permanent gas permeability. The results of the carbon-filled membranes offer perspectives for application in gas/vapor separation with improved mechanical resistance.

## 1. Introduction

Storage and handling of gasoline, and also refueling of cars, involve an outflow of gasoline vapors into the atmosphere [1]. Refueling stations can solve this problem by draining the vapors back into the tanks. The rest of the above mentioned operations, unfortunately, cannot be treated this way, thus, losses of hydrocarbons happen. Hydrocarbons emitted into the atmosphere mean not only an environmental stress, but also financial losses and the waste of energy put into their production. Hence, since the 1980s there was an effort to capture these hydrocarbons and to recycle them. Presently, the process of volatile organic compounds (VOCs) removal from the air is carried out by different methods. The most widely used technique is the absorption of VOCs in activated carbon or in a suitable solvent. Nevertheless, the absorption is a discontinuous process where periodic replacement of the absorbent is needed and, therefore, it is connected with the risk of the rise of toxic waste dumps and wastewater production [2].

In contrast, membrane separation constitutes a safer and more advanced method, and membrane separations for VOCs removal are characterized by a high efficiency. The main advantages offered by membrane processes are [3,4]:

- energy savings;
- environmental friendliness;
- easy handling;
- continuous process;
- compact design and small footprint.

The investment costs of membrane units are higher than in the case of conventional separation methods; hence, the process has to be optimized with respect to membrane area and required quality of purification [5].

Among polymer membranes used for VOCs separations [6], those based on polydimethylsiloxane (PDMS) predominate [7,8,9]. PDMS is a highly efficient organophilic rubbery polymer, which may be applied either supported or as membrane itself. A porous support enhances the mechanical strength of the membrane and enables the use of very thin active polymer layers. Unfortunately, the chemical stability of PDMS is not sufficient and it also swells strongly [10] when it is in contact with organic vapors. Therefore, there is still a search for alternative materials to PDMS with comparably suitable characteristics and better stability. In the past few decades, various membrane materials have been tested, such as, for example, poly(ether-amide) block-copolymer (PEBA), polyvinylidene fluoride (PVDF), high-free volume amorphous glassy perfluoropolymers [11], cross-linked fluorinated or poly(amide-imide) polymers [12,13], and semi-crystalline polyolefins [14].

In flat sheet configuration, membranes are usually subjected to compression forces. These forces may become significant in high-pressure applications, such as in membranes for gas separation or for reverse osmosis. In the case of inhomogeneous porous supports, such compression forces will be translated into a tensile force in the dense skin. Therefore, knowledge of the material’s tensile properties is important. The latter is particularly relevant in the case of hollow fiber membranes, in which the internal pressure is translated immediately into a tensile force on the membrane wall [15].

The appropriate selection of polymer can guarantee sufficient chemical resistance of the final membrane for permeation of gases or vapors. In this context, polyolefines can be considered as potential candidates for membrane applications. The relatively low material cost of polyolefins [16] is also important from the economical point of view. EOC was chosen in the present work as it is more permeable than the semi-crystalline analogous polyolefins polyethylene and polypropylene reported previously [14].

In order to overcome the limitation of both polymeric and inorganic membranes, Mixed Matrix Membranes (MMMs), consisting of a dispersion of filler particles within a polymeric matrix, have been widely investigated to overcome the upper-bound trade-off limit of the polymeric membranes as well as the main drawbacks, such as brittleness and lack of reproducibility associated with inorganic membranes [17]. Thus, these systems are potentially suitable to combine the exclusive advantages in separation performance of both inorganic and polymeric materials. Chemical structure, surface chemistry, size, and aspect ratio are the most important variables for filler selection, whereas filler-polymer compatibility and filler distribution are the key points for an effective MMM preparation [17]. Theoretical models are used to predict and interpret the gas transport properties in MMMs. A basic approach uses the permeability of the two phases and the filler concentration (Maxwell’s model). Some modifications were proposed in order to take into account the filler aspect ratio, as well as the contribution of the interface polymer/filler [18]. Porous fillers are used to enhance transport rates, although it has been demonstrated that dense fillers can also have this effect if the polymer-particle interface plays an important role [19].

Carbon fillers, such as carbon nanotubes (CNTs) and carbon fibers (CFs), are very interesting materials for nanocomposites preparation with a high reinforcing potential, already exploited in different applications (e.g., aerospace and transportation). CNTs or CFs are often added to polymeric matrixes for mechanical reinforcement, and also for an increase of their electrical and thermal conductivity [20]. Alternatively, changes of the electrical conductivity of the composite material induced by exposure to gases and vapors [21], by changing the ambient temperature [22], or by mechanical deformation of the membrane [21,23], make these materials potentially suitable for sensor applications. In the case of membrane separation processes involving combustible gases, enhanced electrical conductivity of membranes improves the overall process safety, preventing electrical charge accumulation. Finally, the electrical conductivity of the membranes is also an indirect measure of the dispersion of the filler in the matrix.

The merit of all above mentioned properties depend substantially on the state of filler dispersion in the polymeric matrix. A blending of fillers into polymer matrices in polymer melt is often used for composite fabrication. Carbon-based materials, such as CNTs or CFs, are generally incompatible with polymers, leading to filler agglomeration in polymer matrices rather than individualization of the filler particles. To enhance the dispersion state of filler, high-energy methods, such ultrasound treatment of the filler dispersed in the polymer solution, are usually used. Better results are achieved by the precipitation of the polymer from solution using a non-solvent [24] to prevent filler sedimentation and aggregation than by the solvent casting method [25]. This may also apply in the case of ethylene-octene copolymer (EOC), which dissolves in cold toluene but is not soluble in common solvents, such as acetone.

In some cases, CNTs and CFs have been reported to improve the transport properties in dense rubbery membranes [26] or in glassy polymer membranes [27]. They have also been used in their neat form as materials for water purification and gas separation membranes [28]. In the present manuscript, the dispersion of these carbon fillers in EOC will be investigated, with particular interest for mechanical properties and the gas and vapor transport properties of the resulting MMMs. The effect of the various carbonaceous fillers on the mechanical properties of the hybrid materials is studied in terms of maximum strength and deformation at sample failure, and in terms of deformation rate-dependence of the elastic modulus at low deformation.

## 2. Experimental Section

### 2.1. Materials

Ethylene-octene copolymer (abbreviated EOC) with 45% octene (ENGAGE 8842) was supplied by Dow Chemicals (Midland, MI, USA). The density of this EOC was 0.8595 g cm^−3^, melt flow index was 1.02 dg min^−1^ (at 190 °C/2.16 kg), and melting temperature *T*_m_ ~ 50 °C [29].

Purified multi-walled carbon nanotubes (MWCNTs), produced by chemical vapor deposition of acetylene were supplied by Sun Nanotech Co. Ltd., Jiangxi, China. Their properties were: nanotube diameter 15 ± 6 nm, length 3 μm, purity of ~90%, density of 1.7 g cm^−3^, and resistivity of 0.12 Ω cm [20]. Further, part of used CNTs was oxidized by nitric acid.

Vapors Grown Carbon Fibers (VGCFs), with trade name VGCF^®^, were supplied by Showa Denko K.K., Tokyo, Japan. Their properties are: diameter 150 nm, length 10 μm, density 2.0 g cm^−3^, and a resistivity of 0.012 Ω cm.

Gases for permeability tests (Pirossigeno, Castrolibero (CS), Italy) all had a purity of at least 99.998%. The solvents hexane, toluene, and ethanol were purchased from Carlo Erba Reagenti (analytic grade, Cornaredo, Italy) and were used without any further purification.

### 2.2. Membrane Preparation

Composites of MWCNT and CF fillers in an EOC matrix were prepared by dispersion of the fillers in the polymer solution, using the ultrasonication method. Firstly, a solution of 5 wt % EOC in toluene was prepared and calculated amounts of fillers were added to yield composites containing 2, 5, 10, 15, 20, 25, 30, and 35 wt % of fillers in the final blend. The sample compositions are given in Table 1, along with the electrical conductivity, which was determined as reported previously [30]. The sonication process was carried out in a thermostatic ultrasonic bath (Bandelin electronic DT 103H, Berlin, Germany) for 4 h at 85 °C. Just after sonication, the dispersions were poured into acetone at room temperature under continuous stirring. Acetone is a non-solvent of EOC and therefore this process led to precipitation of the EOC/MWCNT and EOC/CF nanocomposites from toluene dispersion. The products were then dried under vacuum at 40 °C. Neat EOC membranes and the composite membranes were prepared by compression molding at 100 °C, which eliminates the porosity formed in the material during the precipitation of the polymer/filler composite by coagulation in the non-solvent.

**Table 1:** DC electrical conductivity of the EOC composite samples containing multi-walled carbon nanotubes (MWCNT) or carbon fibers (CF).

EOC/MWCNT				EOC/CF		
wt % of MWCNT	vol % of MWCNT	DC Conductivity (S cm^−1^)		wt % of CF	vol % of CF	DC Conductivity (S cm^−1^)
2	1	(3.16 ± 0.91) × 10^−9^		2	1	(3.48 ± 0.85) × 10^−9^
5	3	(3.25 ± 0.83) × 10^−9^		5	2	(3.54 ± 0.81) × 10^−9^
10	5	(4.08 ± 0.76) × 10^−9^		10	5	(7.54 ± 0.44) × 10^−6^
15	8	(4.61 ± 0.30) × 10^−3^		15	7	(1.46 ± 0.24) × 10^−2^
20	11	(6.32 ± 0.22) × 10^−3^		20	10	(3.13 ± 0.19) × 10^−2^
25	14	(1.34 ± 0.24) × 10^−2^		25	13	(1.60 ± 0.21) × 10^−1^
30	18	(2.53 ± 0.18) × 10^−2^		30	16	(4.28 ± 0.14) × 10^−1^
35	21	(8.62 ± 0.15) × 10^−2^		–	–	–

### 2.3. Membrane Characterization

#### 2.3.1. Thickness and Morphology

The membrane thickness was measured with a digital micrometer (Mitutoyo, model IP65, Lainate, Italy), averaging five measurements. The standard deviation of the thickness of each sample was about 7%. The structure of EOC composites were analyzed by scanning electron microscope (SEM) Vega LMU, produced by Tescan Ltd., Brno, Czech Republic. The samples were cut by Mikrotom Leica RM2265, Brno, Czech Republic, deposited on carbon targets, covered with a thin Au/Pd layer, and observed in the regime of secondary electrons.

#### 2.3.2. Mechanical Tests

Mechanical properties of all membranes were carried out on a Zwick/Roell Universal Testing machine (single column, model Zwick Z2.5, Ulm, Germany) equipped with a 50 N maximum load cell and with pneumatic clamps [31,32]. The clamps surface was covered with an adhesive rubber to avoid slipping of the membrane strips. The membrane samples were cut into strips of 5 mm width. The effective membrane strips length was 30 mm (*i.e*., the distance between the clamps). The strips thickness was measured with the digital micrometer in at least five points and the average value was used.

The membrane strips were stretched to a pre-load of 0.1 MPa before the start of the mechanical tests. The initial speed was 15 mm/min (corresponding to 50% deformation per minute) for the Young’s modulus determination. The test speed was 150 mm/min (corresponding to 500% deformation per minute). The Young’s modulus was determined in the initial linear part of the stress-strain curve between 0.3 and 0.6 MPa. The tensile tester was controlled and the stress-strain curves were recorded and elaborated by the Zwick/Roell Master TestXpert software. The average value and the standard deviation of the Young’s modulus, the tensile strength, and the maximum deformation were determined on a series of four to seven samples.

Further, the deformation rate-dependence of the Young’s modulus was determined using square samples of 3 cm × 3 cm. After clamping, the effective length of samples was 2 cm and the width was 3 cm. As a first reproducibility test, the samples were stretched repeatedly to low deformation (≤10%), making sure to remain in the fully reversible elastic deformation range. The initial crosshead speed was 10 mm/min (corresponding to 50% deformation per minute). The measurement was stopped when a stress of 0.4 MPa was reached. Thus, the Young’s modulus was determined in the initial linear part between 0.2 and 0.4 MPa. The test was repeated ten times, alternating measurements in one direction and in the perpendicular direction to check for heterogeneity in the sample due to preferred orientation of the nanofillers in the flow direction during the melt-pressing. The sample was turned by 90 degrees after each test to avoid possible irreversible plastic deformation in a single direction. Different test speeds were used for the determination of the deformation rate-dependence of the Young’s modulus. The deformation rate was stepwise increased from 1 to 500 mm min^−1^, corresponding to 5%–2500% min^−1^, and then again decreased to 1 mm·min^−1^ to check for possible hysteresis effects. In all cases the maximum stress was kept below *ca.* 0.5 MPa and the total deformation was kept below 35%, well within the elastic deformation regime. To verify the sample homogeneity, the specimen was turned 90 degrees between each measurement.

#### 2.3.3. Gas and Vapor Permeability Measurements

The permeation experiments were performed on a fixed volume/pressure increase instrument [33,34], constructed by GKSS (Geesthacht, Germany). The feed gas pressure was set at 1 bar (the actual value was read with a resolution of 0.1 mbar); the permeate pressure was measured in the range from 0 to maximum, 13.3 mbar, with a resolution of 0.001 mbar. The same protocol was followed for the neat polymer membrane as well as for all hybrid samples.

The gases were always tested in the same order (He, H_2_, N_2_, O_2_, CH_4_, and CO_2_), although it was verified by repeating a measurement cycle that if sufficiently long vacuum was applied to completely remove the previous gas, that the measurement order for these materials was irrelevant. Feed pressure, permeate pressure, and temperature are continuously recorded during each measurement run. The temperature was controlled at a constant temperature of 25 ± 1 °C.

Before the first measurement, the membrane cell was evacuated for sufficient time (at least 1 h) with a two-stage rotary pump. Between two subsequent measurements, the system was evacuated for a period of at least five times the time lag of the previous species in order to guarantee the complete removal of the previous gas. Circular membranes, with an effective exposed surface area of 11.3 cm^2^ (for gas permeation measurement) or 2.14 cm^2^ (for vapor permeation measurement), were used.

Gas permeation measurements were carried out before those with vapors. Vapor permeation measurements were carried out at different feed pressures, ranging from 15 mbar to 200 mbar (*i.e*., vapor permeation measurements were performed at different vapor activities—within the range from 0.3 to 0.9). After the vapor permeability measurements a control experiment with a permanent gas was carried out again to check whether the presence of vapors had altered the membrane properties for the representative samples. This was never the case.

The pressure increase on the permeate side was recorded as a function of time from the moment that the membrane was exposed to the feed gas or vapor. The whole permeation curve takes the following form [34]:

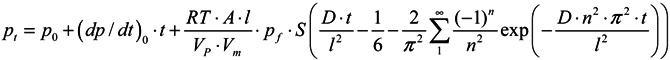
(1)
in which *p*_t_ is the permeate pressure at time *t* and *p*_0_ is the starting pressure, typically less than 0.05 mbar. The baseline slope (d*p*/d*t*)_0_ is usually negligible for a defect-free membrane. *R* is the universal gas constant, *T* is the absolute temperature, *A* is the exposed membrane area, *V*_P_ is the permeate volume, *V*_m_ is the molar volume of a gas in standard conditions (0 °C and 1 atm), *p*_f_ is the feed pressure, *S* is the gas solubility, *D* the gas diffusion coefficient, and *l* the membrane thickness.

The time lag method [35] was applied to the recorded data to determine the gas diffusion coefficient. The permeability coefficient, *P*, is calculated from the following equation, describing the steady state permeation:

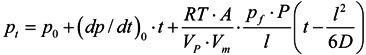
(2)
the last term in Equation (2) corrects for the so-called permeation time lag, Θ, which is inversely proportional to the diffusion coefficient of the gas:

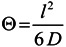
(3)
the gas solubility coefficient, *S*, was obtained indirectly as the ratio of the permeability to the diffusion coefficient by assuming the solution-diffusion transport mechanism:

*S = P*/*D* (4)

permeabilities are reported in Barrer [1 Barrer = 10^−10^ cm^3^ (STP) cm cm^−2^ s^−1^ cmHg^−1^].

## 3. Results and Discussion

### 3.1. Membrane Preparation

The present membrane preparation protocol by melt-pressing of a pre-formed dispersion was used as the conventional solution casting and solvent evaporation method is known to cause often aggregation of the nano-fillers during the evaporation step. By making a solution and quickly coagulating this in a non-solvent, it was found that such aggregation can be avoided for the CNTs and the CFs. In this light, EOC has the advantage over polyethylene and polypropylene of a good solubility in toluene at room temperature. The conditions of the melt pressing are chosen such that the porous structure formed during phase inversion was eliminated, producing completely dense films. The high viscosity in this molten phase guarantees that no aggregation of the fillers takes place during this step. The mutual affinity between the ox-CNTs is, however, so strong that there is already some aggregation in the original solution and, consequently, also in the final films. Silicone coating was needed in this case to fix the resulting pinhole defects before the permeability measurements.

It is widely known that ultrasound may damage polymers, especially in solution [36,37]. This was investigated previously by rheological measurements for the preparation of poly(methyl methacrylate)/CNT composites [38]. Based on this experience, the experimental conditions to prepare the filler dispersions were chosen in such way as to minimize the possible effect on EOC in the present work. As seen from the results of the mechanical tests (see below), in none of the cases the mechanical resistance decreases compared to the neat polymer, confirming that the effect, if any, is smaller than the reinforcing effect of the filler particles themselves.

### 3.2. Membrane Morphology

**Figure 1:**
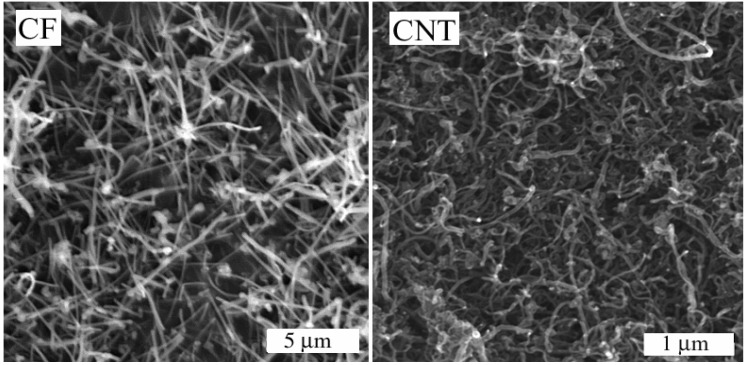
Scanning Electronic Microscopy images of used fillers: carbon fibers (CFs) and carbon nanotubes (CNTs).

**Figure 2:**
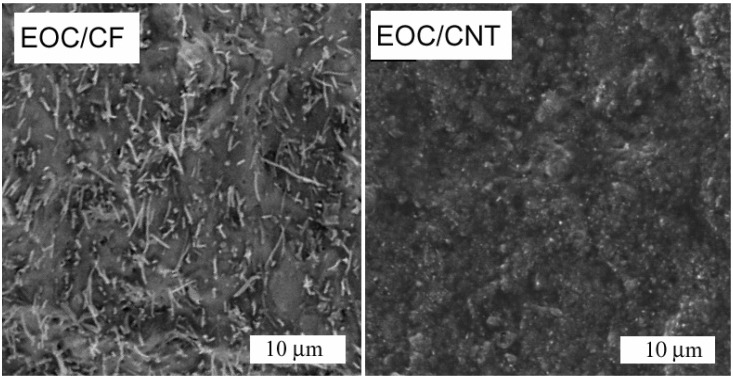
SEM images of fractured surface of EOC/CF composites (20 wt % CF) and EOC/CNT composites (25 wt % CNT).

SEM observations on representative samples of the fillers and the membranes are given in Figure 1 and Figure 2. The samples with oxidized CNTs had a rough surface, indicating poor dispersion of the fillers as large aggregates. The untreated CNTs yielded smooth and homogeneous samples, but the presence of fibrous structures in the SEM images, at relatively low magnification, indicates that the CNTs are present in the form of bundles. The electrical conductivity is given in Table 1. The conductivity sharply increases above approximately 10 wt % of CNT or CF in the EOC matrix. Although the absolute values are lower than those of EOC composites with CNTs functionalized with hyperbranched polyethylene [39], the high conductivity is an indirect confirmation of the good dispersion of the carbon fillers in the polymer matrix.

### 3.3. Mechanical Tests

#### 3.3.1. Stress-Strain Behavior

**Figure 3:**
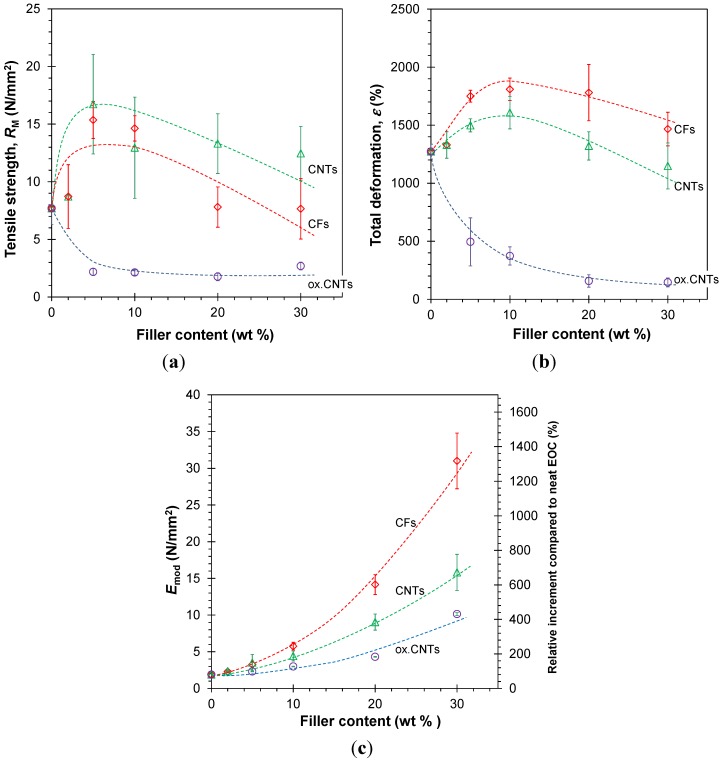
Tensile strength (**a**), maximum deformation (**b**), and Young’s modulus (**c**), as a function of the concentration of CNTs, oxidized CNTs, and CFs in the EOC-based composite membranes.The average Young’s modulus and its standard deviation were obtained from measurements of both strips (4–7 specimens) and square samples (2 specimens). The right axis (**c**) gives the relative increment compared to the neat polymer. Tensile strength and maximum deformation were based on strips only. Lines are plotted as a guide to the eye.

The effect of the different fillers on the elastic modulus of the membranes is plotted in Figure 3. In all cases, the fillers increase the Young’s modulus (*E*_mod_) in an approximately quadratic way.

The Young’s modulus increasing with the fillers addition is expected mechanical improvement. The strongest effect is observed for the carbon fibers, especially at high concentration. The quadratic correlation characterizes the filler-filler interactions giving a second order reinforcing effect [40]. This trend emphasizes the quantitative differences between the three filler types composites. The strong reinforcement results in rubbery membranes with a higher mechanical resistance. The oxidized CNTs show a relatively small enhancement of the elastic modulus due to particle aggregation and poor dispersion in the EOC matrix.

**Figure 4:**
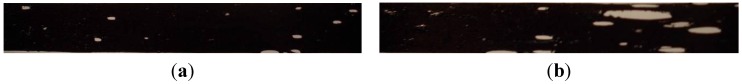
Optical photographs showing the defects during the tensile test of the EOC sample containing 20% of oxidized CNTs. Image rotated by 90 degrees. (**a**) Small deformation; (**b**) Large deformation before rupture.

In contrast to the steady increase of the Young’s modulus with increasing filler content, the tensile strength (*R*_m_), and the elongation at break (ε_max_) of the untreated CFs and CNTs show a maximum. The untreated CFs and CNTs have a similar effect and both fillers increase *R*_m_ at low filler concentration (2%–10%). At higher loading the tensile strength of the CNT reinforced membrane remains twice as high as that of neat EOC and the CF filled membranes, which return to the value of EOC. The elongation at break (ε_max_) shows a maximum in the same concentration range. It then decreases to the value of EOC for the CNTs and remains slightly higher for the CFs-reinforced membranes. In contrast, the oxidized CNTs reduce the tensile strength and the maximum deformation for all compositions due to a poor dispersion and to defect formation in the films (Figure 4).

#### 3.3.2. Frequency Dependence of the Young’s Modulus

**Figure 5:**
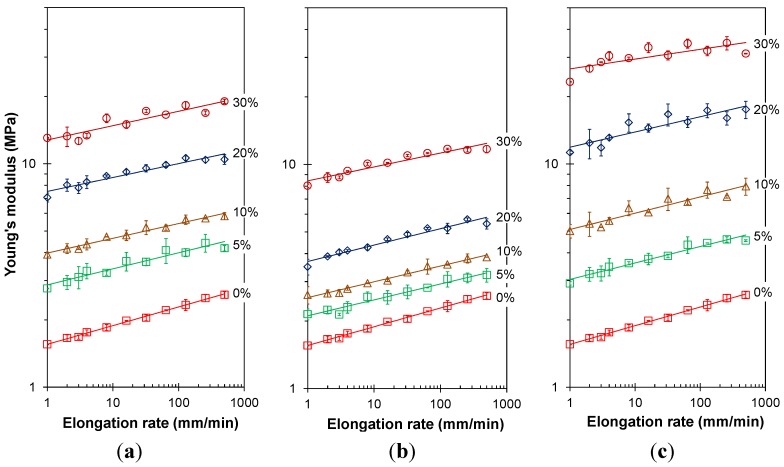
Young’s modulus as a function of the elongation rate for both neat EOC and EOC/CNTs or EOC/CFs composites. Sample length is 2 cm and width is 3 cm. (**a**) CNT; (**b**) ox-CNT; (**c**) CF. The solid lines represent the best fit of the experimental data with the power function given in Equation (5).

The deformation rate dependence of the Young’s modulus is plotted in Figure 5. In some cases, at the highest concentrations of CF and CNT, the deformation rate dependence of the modulus shows a slight zig-zag pattern. As the samples were tested alternatingly with perpendicular orientation, this reflects a slight non-uniformity of the sample, presumably due to flow-induced orientation of the fibers or CNTs in a preferential direction during the melt-pressing of the samples.

**Figure 6:**
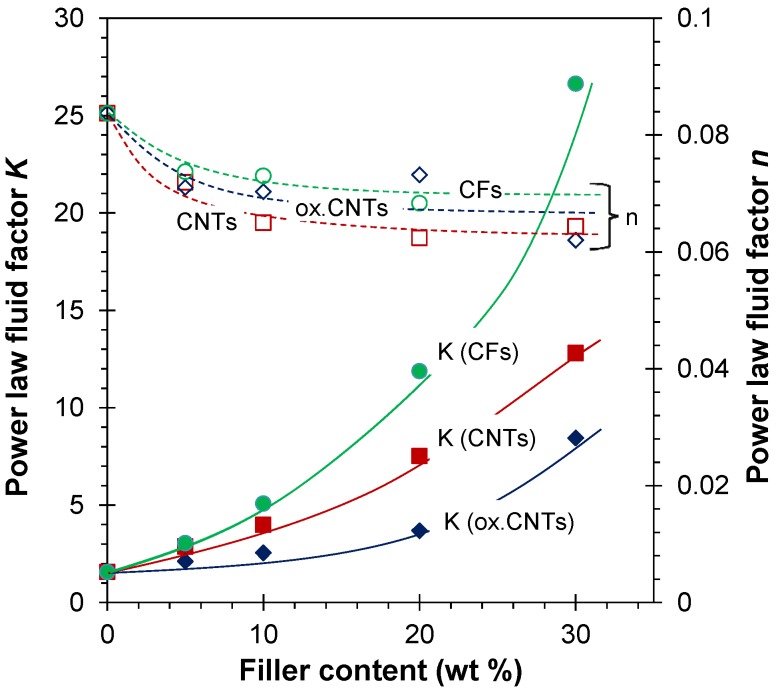
Powerlaw fluid factors *K* and *n*, obtained by fitting the experimental Young’s modulus of the composite membranes with Equation (5) as a function of the filler loading. Lines are plotted as a guide to the eye.

The average Young’s modulus can be described fairly well by a power equation over the entire range of deformation rates of nearly three orders of magnitude:

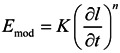
(5)
where *K* and *n* are constants and ∂*l*/∂*t* is the sample deformation rate. The coefficients of Equation (5) are given in Table 2. The value of *K* represents the Young’s modulus at a deformation rate of 1 mm·min^−1^ (5% min^−1^) and increases rapidly with the filler content in a similar fashion as seen in Figure 3, whereas the value of *n* tends to decrease slightly with increasing filler content (Figure 6). Thus, whereas the modulus itself depends strongly on the filler content, the deformation rate dependence of the modulus decreases only slightly with filler content, *i.e.*, the typical viscoelastic behavior is slightly depressed by the presence of stiff, yet purely elastic, fillers.

**Table 2:** Power law fluid factors *K* and *n*, obtained by fitting the experimental Young’s modulus from the tensile tests (Figure 5) with Equation (5).

Filler content (wt %)	Sample							
	EOC + CNTs			EOC + ox-CNTs			EOC + CFs	
	*K*	*n*		*K*	*n*		*K*	*n*
0	1.56	0.0837		1.56	0.0837		1.56	0.0837
5	2.87	0.0719		2.11	0.0710		3.04	0.0737
10	3.98	0.0650		2.55	0.0703		5.08	0.0730
20	7.51	0.0624		3.69	0.0732		11.9	0.0683
30	12.8	0.0644		8.44	0.0620		26.6	0.0437

### 3.4. Transport Properties

#### 3.4.1. Gas Permeation Measurements

**Figure 7:**
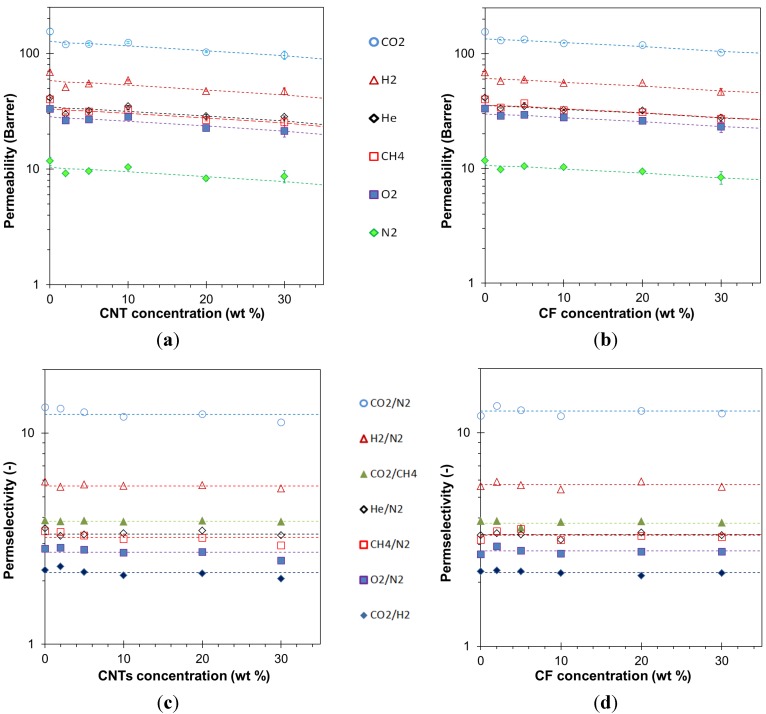
Gas permeability and corresponding ideal permselectivity for EOC/CNTs films (**a**,**c**) and EOC/CFs composite films (**b**,**d**) as a function of the carbon filler concentration. The spread in the data of repeated measurements is of the same order of magnitude as the symbol size. Lines in the permeability graphs correspond to the least squares fit of the experimental data with the Maxwell equation (Equation (6)). The lines in the selectivity graphs correspond to the calculated ratio of the fitted permeabilities.

The gas permeability could be determined directly on the films containing untreated CNT and CF fillers, whereas the films containing ox-CNTs needed a silicone coating to close the pinhole defects. The results of the permeability measurements are given in Figure 7. The most permeable species is CO_2,_ confirming a solubility-controlled transport and the permeation order of different gases is not affected by the filler addition. Although the CO_2_ permeability in EOC is about 20 times lower than that in PDMS [41], it is more than an order of magnitude higher than that in polyethylene and polypropylene [14]. Ideal gas permselectivity, obtained as ratio between permeability of pure species, virtually does not depend on the filler content, whereas the gas permeability slightly decreases as the filler content increases. The same trend was observed for both CNTs and for CFs. The reduction in permeability can be explained if the fillers act as inert, nonpermeable obstacles in the polymer matrix. This behavior can be described satisfactorily by the Maxwell model, which is typically used to interpret the transport properties of mixed matrix membranes [17]:

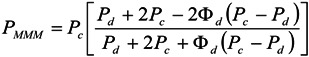
(6)
where *P*_MMM_ is the permeability of the mixed matrix membrane, *P*_c_ and *P*_d_ represent the gas penetrant permeabilities in the continuous and dispersed phase, respectively and Φ_d_ is the volume fraction of dispersed phase.

The applicability of the Maxwell model is interesting because ideally it applies to systems containing low concentrations of spherical fillers. In all cases, the gas transport behavior of the CF-filled membranes is practically identical to that of the CNT-filled membranes. Therefore, only the latter will be described in more detail. The MMM permselectivities were close to that of the neat EOC, suggesting that the copolymer adhered well to the CNTs or CFs and that the corresponding MMMs are defect free. This is further supported by the same order of gas permeability for both neat EOC and filler/EOC composites, with only a modest change in the absolute permeability.

**Figure 8:**
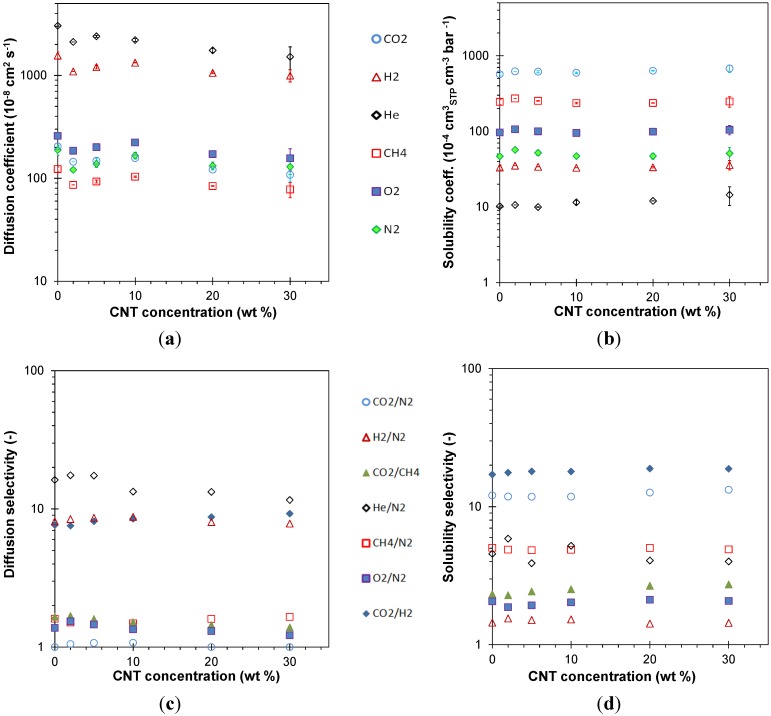
Gas diffusion coefficients (**a**) and solubility coefficients (**b**) with the corresponding selectivities (**c**,**d**) for EOC/CNT films reported in Figure 7 as a function of the CNT concentration. The spread in data of repeated measurements is of the same order of magnitude as the symbol size.

The diffusion coefficients, determined by the time lag method, and the indirectly calculated solubility coefficients are plotted in Figure 8. The trend in the diffusion coefficients closely resembles that in the permeability coefficients and the solubilities appear to be practically independent of the filler concentration. As expected, the smallest molecules, helium and hydrogen, posses the highest diffusion coefficients, both in neat EOC, and in the carbon-filled membranes. In spite of its lower diffusivity, the solubility of CO_2_ is so much higher than that of the other gases that the membranes are nevertheless CO_2_ selective. This is a typical characteristic of rubbery membranes.

#### 3.4.2. Vapor Permeation

**Figure 9:**
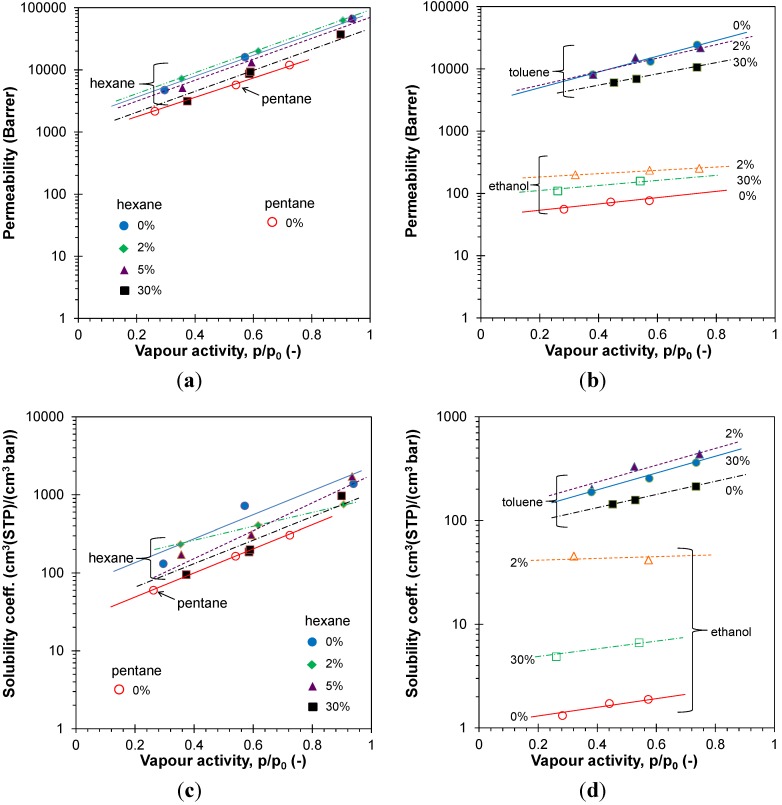
Alkane permeability coefficient of EOC/CNT composite films (**a**) and toluene and ethanol permeability coefficient of EOC/CF composite films (**b**) as a function of the vapor activity; (**c**,**d**) corresponding solubility coefficients, calculated from Equation (4). Lines are plotted as a guide to the eye.

Vapor permeation tests were carried out with representative alkanes (hexane), aromatics (toluene) and alcohols (ethanol) on all membranes. A more extensive series of vapors was used with both the neat EOC membranes and EOC + 10% CNTs. The vapor permeability (Figure 9) is three to four orders or magnitude higher than the values measured with permanent gases. In line with this observation, the permeability and solubility of pentane is inferior to that of the higher alkanes. Both in the neat EOC and in the CNT- and CF-based membranes, the vapor permeability increases significantly with the vapor activity. The same trend is observed for the vapor solubility, confirming a solubility-controlled transport, as generally expected in rubbery polymers. 

The ethanol permeability is about two orders of magnitude lower than that of the alkanes and of toluene and it is also much less activity dependent. This is mainly due to a lower solubility of ethanol in the EOC matrix. Both the CFs and the CNTs significantly increase the permeability of ethanol. This effect seems to be strongest for low filler concentrations. Ethanol permeability is of the same order of magnitude as that of CO_2_ but higher than that of the other permanent gases (Figure 7 and Figure 9). Toluene and hexane permeability are about two orders of magnitude higher compared with the permanent gases. This confirms the potential applicability of neat and hybrid EOC membranes for organic vapor removal from air or from light gases [6].

**Figure 10:**
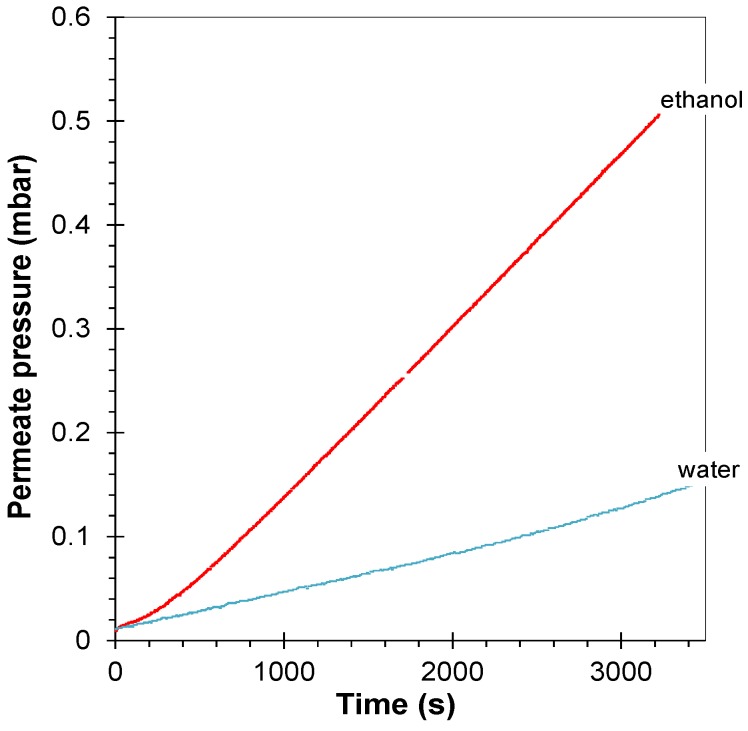
Time lag measurement of a neat EOC membrane with water vapor (*p*/*p*_0_ = 0.65) and with ethanol vapor (*p*/*p*_0_ = 0.57). *T* = 25 °C.

Water vapor permeability was tested in view of possible ethanol/water separation by pervaporation. The ideal selectivity of ethanol/water is 3.0 for the neat EOC (see the time-lag curves in Figure 10). This is somewhat low to be interesting for pervaporation, but clearly shows the organophilic character of the EOC co-polymer, which is more permeable for organic vapors, such as ethanol than for water.

Repetition of the gas transport measurements after vapor exposure of the EOC membranes confirmed that the composites are not affected by the vapors and that no irreversible changes occur in representative samples.

## 4. Conclusions

Gas and vapor transport measurements on ethylene-octene copolymer membranes show that the EOC is an organophilic material that can be potentially used for membrane vapor separation from air or for some gas separations involving mixtures of highly condensable and lighter species.

Addition of carbon nanotubes and carbon fibers has relatively little effect on the transport properties of light gases and a modest effect on the transport of vapors. For light gases, the carbon fillers act as impermeable obstacles and the gas permeability slightly decreases in a similar fashion as predicted by the Maxwell model. For vapors, the behavior is opposite and the addition of a small amount of carbon filler causes an increase in permeability. At the same time, the CNTs and especially the CFs enhance the Young’s modulus of the blends more than ten-fold at the highest loading tested (30%), while both the tensile strength and the maximum deformation present a maximum near the filler content of 10%.

Thus, the carbon fillers enhance the mechanical resistance of the membranes, maintaining or even improving their transport properties. If this behavior is maintained under operating conditions, as may be expected, the presence of carbon fillers will counteract the effect of plasticization of the membranes by vapor sorption, which often compromises the performance of conventional membrane systems. Therefore, the reported membranes are potentially suitable candidates for vapor removal from permanent gas streams.
